# Comparison of Allergic Adverse Effects and Contrast Enhancement Between Iodixanol and Iopromide

**DOI:** 10.5812/iranjradiol.7696

**Published:** 2012-06-30

**Authors:** Farideh Gharekhanloo, Saadat Torabian

**Affiliations:** 1 Department of Radiology, Besat Hospital, Hamedan University of Medical Sciences, Hamedan, Iran; 2 Department of Community Medicine, Hamedan University of Medical Sciences, Hamedan, Iran

**Keywords:** Iodixanol, Iopromide, Hypersensitivity

## Abstract

**Background:**

Iodinated X-ray contrast media are the most commonly used contrast agents in the world with an annual application of 40-50 million. New non-ionic contrast agents are subdivided into low osmolar agents such as iopromide and iso-osmolar agents such as iodixanol. Regarding different biochemical characteristics, these agents are different in the allergic reactions and contrast enhancement and final lesion conspicuity.

**Objectives:**

This study was carried out to compare allergic adverse effects and contrast enhancement between iodixanol and iopromide.

**Patients and Methods:**

One-hundred and twenty patients who were referred for abdominal CT scan to Besat Hospital were included in this study. Patients were randomly divided into two groups (A and B). Group A received 100 cc iodixanol (300 mgI/mL) and group B received 100 cc iopromide (300 mgI/ml) by power injector. CT examination was performed using Helical CT Scanner (Somatom, Siemens, Germany). Sixty seconds after injection, images were obtained and enhancement of port, liver and aorta were determined. Allergic adverse effects were recorded one hour and up to one week after injection.

**Results:**

Iodixanol produced a significantly greater enhancement of the hepatic, aorta and portal vein than iopromide (P < 0.01). Sixty seconds after injection, associated pain and heat sensation were less frequent in iodixanol in comparison with iopromide (P = 0.03). Immediate reactions such as nausea and vomiting were less frequent in iodixanol (P = 0.01). Late skin reactions such as rash was more frequent in iodixanol (P < 0.01).

**Conclusions:**

Iodixanol is safe and is better tolerated in the early phase of injection with better contrast enhancement and lesion conspicuity. Mild late skin rash is its disadvantage.

## 1. Background

Iodinated X-ray contrast media are the most commonly used contrast agents with an annual worldwide application of 40-50 million. They are subdivided into four groups; namely, ionic monomer, ionic dimmer, non-ionic monomer and non-ionic dimmer. New non-ionic contrast agents are subdivided to low osmolar contrast media such as iopromide (as a monomer) and iso-osmolar agent such as iodixanol ( as a dimmer ) (1). Two important factors in the use of contrast media are efficacy and tolerability (2). A major factor in the toxicity of ionic monomers is their high osmolality responsible for injection site pain, heat sensation and nausea (2). This toxicity is less common in new non-ionic contrast agents. Allergic drug reaction is divided into immediate and late reaction (3). Immediate reaction occurs up to one hour after injection and is directly related to the osmotic load or to the contrast media chemotoxicity. Late reaction is associated with T lymphocyte-mediated delayed hypersensitivity. Iodixanol and iopromide are most commonly used for imaging purpose in our country and are both available, although to our knowledge, there has been no documented trial study which compares the effectiveness (contrast enhancement) and adverse effects of these routinely used contrast agents (3). In this clinical trial study, we compared the allergic adverse effects and contrast enhancement of iopromide (as a monomer) with iodixanol (as a dimmer).

## 2. Objectives

The aim of this study was to compare the allergic adverse effects and contrast enhancement of low osmolar contrast media (iopromide) with iso-osmolar contrast media (iodixanol).

## 3. Patients and Methods

One-hundred and twenty patients who were referred for abdominopelvic CT scan were enrolled into this study. Patients were allocated into two groups (A and B) based on the block randomization method with block sizes of four. Each group received a different intravenous contrast agent. Group A received 100 cc iodixanol 300 mgI/ mL (visipaque, GE Healthcare) with an osmolality of 290 mOsm/kg. Since 300 mgI/mL concentration of iodixanol was not available, 320 mgI/mL ioxianol was diluted with isotonic saline to a concentration of 300 mgI/mL. Patients in group B received 100 cc of iopromide 300 mgI/mL (ultravist 300, Bayer Schering Pharma) with an osmolality of 620 mOsm/kg. Exclusion criteria were severe fatty liver, hepatic tumor and portal vein thrombosis. CT examination was obtained using a helical CT scanner (Somatom, Siemens, Germany). Injection was carried out through the antecubital vein by an 18 gauge needle. A volume of 100 cc of contrast media was injected at a flow rate of 5 cc/s using a power injector. Sixty seconds after beginning the injection, helical CT sequences were obtained. For determination of hepatic enhancement value, at least three measurements in different areas were obtained and recorded. Enhancement of the aorta was recorded in the same slice hepatic attenuation was measured. Portal vein attenuation was obtained at bifurcation. Immediate allergic adverse effect was recorded up to 1 hour after injection. Patients were followed for 1 week and delayed adverse effects were also recorded. Chi square and t-test were used for statistical analysis.

## 4. Results

**Figure 1:**
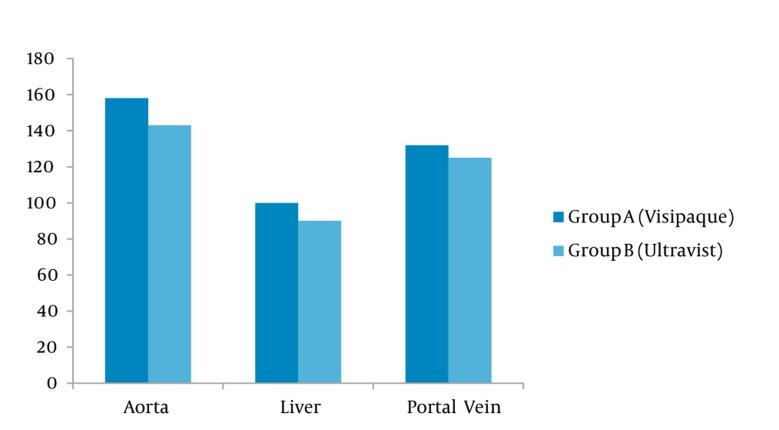
Comparison of contrast enhancement between the two groups

A total of 120 patients were included in this study. There were 49% male and 51% female in group A and 54 % male and 46% female in group B (P = 0.6). The mean age of the patients was 46.3 ± 12 years in group A and 42.2 ± 15 years in group B showing no statistically significant difference. Immediate and late allergic reactions were less frequent in iodixanol in comparison with iopromide (35 % and 23 % vs. 21 % and 16 %, respectively) (Table 1). Injection-associated pain and heat sensation was less frequent in the iodixanol group in comparison with the iopromide group (8.1 % and 5.7 % versus 12.3 and 8.9 %, respectively) demonstrating a statistically significant difference (P = 0.03). Among the late allergic reactions, skin reaction, itching and rash were more frequent in iodixanol (7.9 % and 14.3 %) vs. iopromide (3.5 % and 1.8 %), respectively (P < 0.01). In comparison of contrast enhancement 60 seconds after injection, iodixanol produced a significantly greater enhancement of the hepatic vein, the aorta and the portal vein than iopromide (97.9 HU, 157.8 HU and 131.5 HU vs. 86.9 HU, 143.9 and 119.8 HU, respectively). This value was statistically significant (P < 0.01) (Figure 1).

**Table 1:** Comparison of Immediate and Late Adverse Reactions Between the Two Groups

	**Group A, No. (%)**	**Group B, No. (%)**	***P*value**	**Group A, No. (%)**	**Group B, No. (%)**	***P *value**
Nausea	3 (4.8)	13 (22.8)	0.5	4 (0.7)	8 (14)	0.07
Vomiting	3 (4.8)	5 (8.9)	0.3	1 (1.6)	4 (0.07)	0.1
Abdominal Pain	1 (1.6)	5 (8.9)	0.06	1 (0.6)	5 (8.8)	0.07
Injection Pain	5 (8.1)	7 (12.3)	0.4	0	1 (1.8)	0.2
Injection Hotness	3 (5.7)	5 (8.9)	0.5	0	0	0
Injection Redness	0	0	0	0	1 (0.8)	0.2
Itching	0	0	0	5 (7.9)	2 (3.5)	0.3
Rash	0	0	0	9 (14.3)	1 (1.8)	0.01
Headache	6 (9.5)	13(22.8)	0.04	4 (6.3)	8 (14)	0.1
Dizziness	1 (0.6)	7 (12.3)	0.01	5 (8.1)	5 (8.8)	0.8

## 5. Discussion

Allergic adverse effects are defined as immediate and late adverse reactions. Osmotic load or chemotoxicity is responsible for the immediate reaction. Late reaction is related to T lymphocyte-mediated delayed hypersensitivity (3). In our study, immediate reaction was seen more frequently in iopromide than iodixanol which is an isotonic agent (35 % vs. 23 %). In a study conducted by Albrecht et al., allergic adverse reaction was seen in 16.9 % of the iopromide group (4). In our study, among the late reactions, only skin reactions such as rash were seen more frequently in iodixanol (14.3 %) than iopromide (1.8 %). Verow et al. also reported that in comparison with monomeric contrast media, dimmeric agent causes a higher rate of skin reaction and fever (5). In their study, early discomfort with iodixanol was far less frequent in comparison with iopromide and heat sensation was less intense and pain was less frequent and they reported that iodixanol is as effective as iopromide and produces less discomfort other than the late skin reaction (5). Sutton et al. also reported that visipaque is well tolerated in the early phase of injection (6). In a study by Gomi et al., immediate reaction was reported in 2.7 % in iodixanol in comparison with 3.5 % in iopromide (7). Skin reaction is mostly mild skin reaction which will be resolved simply. However, according to our findings, iodixanol in the early phase of injection offers better comfort and better compliance of the patient. Nausea and vomiting after injection of iopromide practically caused many problems in the process of injection and imaging. In our study also most reactions were mild and we did not have any severe reactions in any of the groups. In a double blind, randomized clinical trial which was carried out by Manke et al., pain and heat sensation in iodixanol was less frequent than iopromide (4 % vs. 9.5 %, respectively) (8). In our study, the same result was obtained. In the study performed by Martin et al., adverse effects were less frequent in iodixanol (1.8 %) compared to iopromide (2.4 %) (9). In our study, the same result was obtained. However, according to the results of Martin et al.’s study, iodixanol has theoretical advantage without any obvious practical benefit (9). In the comparison of contrast enhancement between iodixanol and iopromide, Graf et al. compared contrast enhancement during the first helical CT sequence (30 seconds after injection) and no significant differences were observed between the groups. However, in the second helical CT acquisition (such as our study) significant differences were observed. They believe that during early scan, enhancement difference related to diffusion of contrast or interstitial fluid may not have enough time to become significant. However, during the second helical CT acquisition, there is enough time for diffusion of contrast media between the extracellular fluid and vascular compartment. Some possibility for enhancement improvement of iso-osomolar agents such as iodixanol are as follows: decreased movement of extracellular fluid osmotically as well as efflux of the contrast agent, resulting in a higher vascular and hepatic contrast level and an improved vascular enhancement and also hepatic enhancement (10). In addition, there are studies that have approved 60 seconds for optimum enhancement and diffusion of the contrast between the blood pool compartment and interstitium (11, 12, 13).
